# On not being able to read: doomscrolling and anxiety in pandemic times

**DOI:** 10.1080/0950236X.2022.2056767

**Published:** 2022-03-29

**Authors:** Laura Salisbury

**Affiliations:** English and Film, University of Exeter, Exeter, UK

**Keywords:** doomscrolling, anxiety, time, care, waiting, COVID-19 pandemic, Don DeLillo, W. E. B. Du Bois

## Abstract

This article analyses ‘doomscrolling’, or the compulsive reading of anxiety-inducing online content during the COVID-19 pandemic, against the common idea that it is simply an addictive social practice that impedes mental flourishing. Instead, in order to open up its inclination towards care, I read doomscrolling through the anachronistic neologism that has come to define this specifically textual practice. Tracing the operations that doomscrolling and anxiety perform on lived time, the article uses the work of Eugène Minkowski, Sigmund Freud, Lauren Berlant, Walter Benjamin, and Lisa Baraitser to examine how these practices hope to take care of time when narratives of progressive history have worn thin. I include analyses of the anxious textuality of Don DeLillo’s *The Silence* and Saidiya Hartman’s reworking of W. E. B. Du Bois’s ‘The Comet’ to demonstrate how doomscrolling emerges from a moment when trust is anxiously fractured, but how it works, nevertheless, to witness what gets to count when time is felt to be coming to an end.

I have of late, – but wherefore I know not, – lost all my mirth, forgone all custom of exercises […]

Shakespeare, *Hamlet*, II, I.

Lately, though it hasn’t been clear to me exactly why, I have lost my ability to read. But as soon as I write this it doesn’t seem quite right. I have probably been reading more words than ever, but although keeping abreast of modern and contemporary fiction is one of the things I am paid to do, I have struggled to focus my attention. Through 2020 and 2021, as millions have waited behind the ‘frontline’ of the response to COVID-19, I have found it increasingly hard to contain or retain my reading. My tally of words has accumulated via a compulsive ‘doomscrolling’ of anxiety-inducing social media content and 24-hour online news that has felt both like a distraction and peculiarly driven rather than drifting. And whatever drive has been at work, it has seemed to break up the capacity to place myself in sync with something like a literary novel. Sometimes doomscrolling has been prompted by a sense of something rotten in politics, or an idea of the air as ‘a foul and pestilent congregation of vapours’, or the thing Hamlet doesn’t name but nevertheless suffuses his speech – grief.^1^ But what is the function of this particularly anxious mode of reading that became bound up with the waiting time of the pandemic? And why have Don DeLillo’s short literary fiction *The Silence* (2019) and Saidiya Hartman’s online critical reworking on W. E. B. Du Bois’s ‘The Comet’ (2020) seemed readable when other texts have not? Is it simply that both are thematically concerned with a hiatus in the rhythms of historical time caused by catastrophic events, or is there also something in their anxious textuality that is revealingly aligned with doomscrolling’s affordances?

To approach these questions, I will analyse doomscrolling – an *OED* word of the year in 2020 and clearly what Eve Kosofsky Sedgwick would call a ‘paranoid’ mode of reading –^2^ against its dominant framing as an addictive social practice determined by the monetisation of attention and an obstacle to mental flourishing. Instead, I want to think about it as a symptom, in the psychoanalytic sense of an individual psychic solution to a conflict that has a latent orientation away from what is experienced as intolerable. But I also want to explore whether this specifically textual practice might be an attempt at a more ‘social cure’, which uses the animating force within reading as a ‘reparative’ orientation towards the renewal and rebuilding of connections between people and things in the world that feel increasingly frayed, even ruptured or broken.^3^ Cure is perhaps the wrong word, though. For the symptom’s attempt to mitigate distress is never exactly an orientation towards health; instead, it might better be understood as an inclination towards a certain kind of care of the self. To put it differently, I am wondering whether this mode of anxious reading while waiting might be an attempt to ‘*maintain, continue, and repair our “world” so that we can live in it as well as possible*’, to use Berenice Fisher and Joan Tronto’s famous definition of care,^4^ even as doomscrolling is more obviously associated with care in its older sense – worry, lament, sorrow.

## Anxious times

The worst is not So long as we can say, ‘This is the worst.’

Shakespeare, *King Lear*, IV, I.

Since the beginning of the COVID-19 pandemic, it has become commonplace to note how these times, monotonously framed as ‘extraordinary’, have produced significant changes in people’s lived experience of temporality. Lockdowns and shelter in place orders; social (or spatio-temporal) distancing; the feelings of fear and grief as deaths mount: all have profoundly altered individual and social senses of time, forcing disruptions of work, leisure, and the repetitions and rituals of the social that have produced and exacerbated mental distress for many. The losses associated with the pandemic and its restrictions have also brutally intensified health inequalities and the compounding injustices endured by communities and individuals denied access to many of the resources that could mitigate distress by containing and managing vulnerability.

One key mental and somatic experience of the waiting time of the COVID-19 pandemic, but that has also has seemed insistent in the collective mental life of late liberalism over many years, has been anxiety. Etymologically, ‘anxiety’ is derived from the classical Latin *anxietas* meaning ‘worry, solicitude, extreme care, over-carefulness’ (*OED*). But, in its earliest uses in modern English in the 15th century, the word takes on a particular association with temporality: anxiety is determined by a ‘worry over the future or about something with an uncertain outcome; uneasy concern about a person, situation, etc.; a troubled state of mind arising from such worry or concern’ (OED). Clinically, anxiety emerges in the latter half of the nineteenth century, mostly figured as a sub-set of symptoms that existed alongside and within neurasthenia and melancholia. In 1980, however, the *Diagnostic and Statistical Manual 3* introduced a distinct category of disorders associated with ‘Excessive anxiety and worry (apprehensive expectation)’. And the current *DSM-5* (2013) makes an explicit association not only with the time of the future but with the chronicity of an ongoing condition.^5^ It also distinguishes importantly between anxiety as ‘anticipation of future threat’, and fear, which is ‘the emotional response to real or perceived imminent threat’.^6^ As Joanna Bourke puts it: ‘in fear states, individuals are consciously able to take measures to neutralise or flee from the dangerous object, while purposeful activity fails individuals whose subjective experience is anxiety’.^7^ When anxiety is something that troubles the clinic, then, it is defined by excess: its carefulness is too much in relation to the object; it also goes on for too long. Activities associated with care and paying attention continue, but measured purpose drops away.

In his 1933 book, *Lived Time*, the phenomenological psychiatrist Eugène Minkowski argued that most ‘psychopathologies’ are fundamentally grounded in a disturbance of temporal duration – the organic flow of retentions from the past and projections into the future that suffuse every lived present moment. He wrote positively of ‘activity’, which is braided with the qualities and expression of life itself – a time that can be seized and used. But in ‘expectation’, ‘we live time in an inverse sense; we see the future come toward us and wait for that (expected) future to become present’:

[Expectation] englobes the whole living being, suspends his activity, and fixes him anguished […] It contains a factor of brutal arrest and renders the individual breathless. One might say that the whole of becoming concentrated outside of the individual swoops down in a powerful and hostile mass, attempting to annihilate him; it is like an iceberg surging abruptly in front of the prow of a ship, which in an instant will smash fatally against it. Expectation penetrates the individual to his core, fills him with terror before this unknown and unexpected mass, which will engulf him in an instant. Primary expectation is thus always connected to intense anguish. It is always anxious expectation […] In the presence of an imminent danger we wait, frozen in place as if paralyzed by terror.^8^

In this anxious state, there is little sense of moving through time; rather, the present is overwhelmed and absorbed in an experience that comes to resemble extreme melancholia. For in depression the future also seems ‘blocked by the conviction of a destructive and terrifying event’:

No action, no desire emerged which, emanating from the present, could go toward the future across this succession of dull and similar days. Because of this, each day had an unusual independence. They did not vanish into the sensation of the continuity of life. Each one emerged as a separate island in the dark sea of becoming.^9^

‘[T]ransformed into a succession of similar days unfolding in a boundless sadness or monotony’,^10^ time is spatialiased in a way that echoes Henri Bergson’s famous description of linear time imagined as beads on a necklace.^11^ But such spatialisation isn’t merely an inaccurate account of lived time, as it is for Bergson; for Minkowksi, the fundamental deformation of time’s organic character produces intense mental distress for a self wrecked and washed up far from the flow of duration. Dependent relationships, both on the future and, crucially, by implication, on others, dwindle defensively into islands of diminished, brutally self-contained insufficiency. One languishes rather than flourishes.

But if depression attempts to care for the anxious self by wresting it from imagined destruction as the future engulfs the present, Minkowski also observed compensatory responses to depression’s temporal arrest in ‘the obsessive tendency to count, to check, to ruminate, to follow the most insignificant events of either external or internal life’.^12^ Such attempts to reanimate the present in the face of a ‘pathological slowing-down of time’ only produce an illusion of duration, however: a ‘mechanical progression’ of non-stop cycling in which time doesn’t flow any more than it did in the insularity of depression.^13^ Caught in an alternation of depression and anxious expectation, duration falters and it feels impossible either to live or to die.

Minkowski concentrated only on the future and the present in his account of traumatic ‘expectation’, but in 1926 and in the wake of World War One Sigmund Freud described anxiety as determined by a traumatic structure laid down in the past. He claims that anxiety is a form of unconscious learning from traumatic experience; it recognises from the past the shape of future trauma and functions as a signal that enables defences to be mobilised. Freud writes of signal anxiety:

‘the present situation reminds me of one of the traumatic experiences I have had before. Therefore I will anticipate the trauma and behave as though it had already come, while there is yet time to turn it aside’. Anxiety is […] on the one hand an expectation of trauma, and on the other a repetition of it in a mitigated form.

Alerted by anxiety, the individual can ‘foresee and expect a traumatic situation […] instead of simply waiting for it to happen’.^14^ More recent cognitive psychological models of Generalised Anxiety Disorder concur that ‘[i]ndividuals with GAD tend to execute worrying as a means of coping with anticipated danger’.^15^ But as the anticipated danger does not come to pass, the problem-solving of worrying is associated with having staved off disaster and becomes a repeated coping strategy. This can lead to an intensifying cycle of mental distress as worrying itself, especially in relation to situations that are not obviously fear-inducing, starts to be ‘appraised as uncontrollable and dangerous’.^16^

In both psychoanalytic and cognitivist models, then, anxiety is figured as taking a certain care of the self by producing a particular sense of time. Signal anxiety mitigates helplessness by enabling the psyche to feel that ‘there is yet time’ to avoid disaster, perhaps to retreat into the stilled time of depression, but also to problem-solve through worrying while waiting in a safer, more sheltered place. Although waiting can produce anxiety, the repetitions and obsessive compulsions of anxiety also seem aimed at producing a world in which there would be time enough to wait – a ‘yet time’ where annihilation could be avoided and another kind of future made or at least momentarily imagined. Anxiety represents an attempt to manage threat by proroguing it; still, producing ‘yet time’ always risks a pained or panicked sense of being stuck in the interminable.

## Waiting for Domesday

What’s happening [?]

*Twitter*

As many around the globe have lived through repeated brutal waves of COVID-19, a particular sense of anxious waiting has emerged for those behind the front lines but in close contact with how futures come to feel cancelled. In *Ugly Feelings*, Sianne Ngai reads anxiety as paradoxically aligned with both ‘the temporal dynamics of deferral and anticipation’.^17^ And many now know more than a little of the anxious desire to defer and avoid a catastrophe to come through checking and counting, while at the same time inviting in feelings of disaster through apocalyptic fantasies that serve a psychic purpose in promising to bring the interminable repetitions of ‘yet time’ to an end. In his autobiography, Leonard Woolf described the wartime psychology of September 1939 as markedly different to that of August 1914:

People of my generation knew now exactly what war is – its positive horrors of death and destruction, wounds and pain and bereavement and brutality, but also its negative emptiness and desolation of personal and cosmic boredom, the feeling that one is endlessly waiting in a dirty, grey railway station waiting-room, a cosmic railway station waiting-room, with nothing to do but to wait endlessly for the next catastrophe.^18^

Perhaps many also wait differently now to how they waited in the first wave of COVID-19, with a less fantasised sense of the ‘positive horrors’ of the pandemic, but with a clearer sense of the negative emptiness of living on in its wake.

Time has slipped its usual linear markers. Very many wait in the wake of an ever-growing accumulation of experiences that have certain affective distinctions, and that differ in terms of their intensity (this is not a time of indifference), but that cannot be figured through progressive temporal modes such as those produced by dialectical relationships. As Mathew Arthur puts it: ‘Pandemic short-circuits flows of goods, bads, and bodies. Its ethics are diffuse. Everywhere impinges. Time feels caught between aftermath and looming recurrence: impasse, interruption, repeat’.^19^ Woolf’s image of a ‘grey railway station waiting-room’ invokes cigarette ash, dulled palettes, and the smog of coal and steam of mid-century Britain, while Arthur’s glitched flows evoke unstable connections suspended in the blue-grey light of the digital. But they come together in a shared idea that grey has a relationship to time. If we think of colour as a non-spatial way of figuring time, because grey has no dialectical opposite but is achromatically composed of black and white in various shades of intensity,^20^ grey time might interrupt feelings of contrast that support sensations of temporal progression. Neither simply black nor white nor coloured with stable or easily determinable affect – crisis reds or bluesy longing – grey time speaks instead to living through differences in intensity that gather, but not quite the differences in kind that would seem to allow time’s passage.^21^ Impasse, interruption, repetition.

For Algirdas Greimas and Jacques Fontanille, anxiety itself has a fundamental kinship with grey. Merely tracking an oscillation between basic attraction and repulsion, by their account anxiety is a state that precedes and suspends the polarisation of either euphoria or dysphoria; it therefore ‘prevents the formation of “valencies” and of all definite orientation’.^22^ Greimas and Fontanille quote Camus’s description of the Zuider Zee as a landscape that dissolves valuation: ‘See on your left that pile of grey ashes they call a dune here, the grey dike on your right, the ashen shore at your feet and, before you, the sea that is the colour of a weak detergent’. In this ‘indefinite flatness’, ‘everything is lost in the stagnation of grey engulfment’. Significantly, such states of ‘non-articulated tensions’ have the effect of suspending a movement towards the designation of value, or what Greimas and Fontanille call *fiducia* – trust or confidence in which things can be accounted for.^23^ Ngai argues, following Freud, that anxiety seems fundamentally more aversive than Greimas and Fontanille imply;^24^ but whether anxiety is the suspended grey time of stagnation that precedes affective orientation, or a swerve away from flat indifference into shades of difference that gather rather that pass because the object of anxiety cannot be brought to consciousness and accounted for, anxiety speaks to a suspension of solid valuation. As cognitive psychologists suggest, if worry is a response to anxiety that attempts to problem-solve, to get things sorted into their appropriate places, these insufficiently differentiated repetitions nevertheless struggle to bring anxiety into a state of fear in which action could ‘neutralise or flee from the dangerous object’.^25^ Instead, the anxious person feels either smothered by that which cannot adequately be differentiated or stuck in the grey of depressed avoidance.

Of course, in the social and historical reality of a pandemic there are always differences and distinctions to be made. Black and white are not merged in shared uniformity. People wait and have waited through conditions that are differentiated over time and space: for the numbers of infections and deaths to peak or to drop; for governments to institute measures that might slow the spread of the virus or mitigate the economic effects of non-pharmaceutical interventions; for vaccinations to be developed, rolled out, and shared (or not); for the most basic care; for the operations of state violence and negligence to do their work. These experiences play out in radically uneven ways, whether acutely or chronically. We wait for news of deaths that do not touch everyone equally. We bear experiences of selves or others in sickness that are distinct in their intensity and proximity. We endure through time in material conditions that may be profoundly impoverished or reasonably comfortable. And yet, one thing shared by many who are online but not on the front line – those compelled to wait rather than act – is the reading practice of making digital contact with a glut of information, but an ongoing and radical uncertainty about what such data tell us. Both driven by and withdrawing from the possibility of fi*ducia* in its repetitions of problem-solving and avoidance, the grey time of anxiety does not flow towards a future into which one feels one can step. In the face of the impossibility of making things hold, anxiety instead causes time to stick.

What does it mean, then, to be trying to understand a relationship to the future that feels simultaneously ‘cancelled’ and increasingly anxiously threatening? And what does it mean to attempt such understanding in an environment where there is a surfeit of information but a general fracturing of trust in how such information is gathered and communicated? One of the things it might lead to is doomscrolling. As Mathew Arthur describes it:

Doomscrolling is compulsive swiping through pandemic-related headlines, graphs, and tweets. It is an urge that practices the body into dystopian feeling: an affective becoming-indebted to the way things are through gesture – or a bad news world interfacing the body as a tic [… ] Doomscrolling is killing time as an investment in feeling on top of things – a feedback loop of testing for encroachment. How close, how long, how much, how bad? Again. It mistakes use patterns, forms of connectivity, and attentional modes for reality as a symptom of its own debt.^26^

Arthur captures the atmosphere of compulsive reading and its peculiar braiding of the urge to quantify (how close, how long, how much?) with a qualitative judgment that is experienced affectively (how bad?). How does one register and quantify the encroachment of loss or the proximity of disaster to the self, while also keeping a qualitative connection with catastrophic losses that, in the moment of doomscrolling, are primarily registering in the bodies and lives others? Is there a way of keeping loss in its place, while also making it count?

As Arthur suggests, doomscrolling, not unlike anxious worrying, has the effect of seeming to kill time; but it also makes time of a particular kind in its rolling and roiling. This paradox might be untangled by paying attention to the suggestively anachronistic neologism that has settled on this particular mode of anxious reading. The doom in doomscrolling most obviously evokes death, destruction, or some other ‘positive horror’. From this point of view, doomscrolling is an attempt to reassure oneself that doom, or the worst, is not yet here. But doom is derived from the Old English dom, which means a statute, law, or enactment. The Domesday Book was the twelfth-century name, retrospectively applied, to the ‘the record of the Great Inquisition or Survey of the lands of England, their extent, value, ownership, and liabilities, made by order of William the Conqueror in 1086’ (*OED*). Here, dom signified a final reckoning against which there was no appeal. The question of ‘how much’ was answered, as in the Last Judgment itself. To doom someone is still to pronounce sentence on them, just as the Ghost of Hamlet’s father is ‘Doom’d for a certain term to walk the night’.^27^ But the contemporary sense of fated destruction tracks in the long shadow of the Christian notion of doomsday as the dissolution of all worldly things, of all quantities, but the preservation of certain gathered qualities: the moment when God renders final judgment on the world and all debts are paid. Following the harbinger of plague and pestilence, time itself comes to an end on doomsday. God ‘calls time’, as it were, on the quantity and quality of time itself.

Doomscrolling is not simply a kind of directionless, indefinite oscillation, then. Instead, as Arthur notes, it is a kind of killing time that has an orientation. Yes, we doomscroll to test encroachment and threat, to produce a sensation of time in which we might yet turn aside from a threat that is bearing down. But we also doomscroll to try to manage the ‘negative emptiness’ of a world seemingly without qualities, using online stimulation as an attempt to jump start time into passing in conditions where it feels stuck. We doomscroll in the hope that the world itself will have moved forwards even if we have stayed still. We click through wormholes, or even down rabbit holes, in the hope that a different space might open up; but we return to find that only time has passed, although without much sense of spatio-temporal progression. We also doomscroll as a form of action when compelled to stay still, to become a bystander, with the guilt that entails. In the time of waiting, doomscrolling becomes a practice that attempts to attach judgment and attention to the world, so that things might come to count in a way that can be settled.

It is worth emphasising that scrolling is specifically a reading practice rather than another form of spending time on the internet, such as swiping through memes and images, or surfing and clicking on videos. Many early theorisations of digital reading concentrated on its utopian possibilities; for example, George P. Landow’s foundational 1992 work on hypertext focused on the de-centering of authority and presence (reading alongside Derrida) and the emergence of a ‘readerly’ text (echoing Barthes).^28^ By this account, hypertext enables ludic and creative reading practices that articulate more distributive and unpredictable lines of authority than the fixity of the analogue. But the later emergence of chatrooms, blogs, social media, and rolling news, supported by smartphones and increased speed of access, capitalised on the instability of text that can be overwritten, reshaped, can tick away, and can appear in one’s ‘feed’ semi-automatically and according to algorithmic logics. As new technologies and platforms fashion new configurations of power, the emphasis on reader experience as ludic, in the sense of following the shape of non-end-oriented play, has shifted significantly. Indeed, as gaming and the psychological reward systems of gambling have become absorbed into web, software, and hardware design,^29^ users, and here specifically readers, have been reimagined less as playing than as ‘played’.

Within such a context, and in a post-truth environment in which facts have become radically uncertain – sometimes read as complex social constructions produced by particular matters of concern, sometimes as deliberate attempts to deceive – there may be a particular desire to settle accounts via textual ordering and according to a form of dispositive judgment mobilising an atemporal idea of truth. The judgment offered on Doomsday will not be finessed or overwritten for ideological ends; instead, the ‘issue of this judgment shall be a permanent separation of the evil and the good, the righteous and the wicked’.^30^ Doomscrolling indeed hopes to secure understanding that will have a temporal permanence and will not give way under the pressure of later rhetorical insistence. COVID-19 is indeed a pandemic. Excess deaths show that this isn’t simply a bad flu season. Hospitals really are running out of oxygen. COVID deaths have passed 100 thousand. Certain nations and communities are suffering acutely. The votes have been counted. The Capitol has been stormed.

Doosmcrolling articulates the distance, both spatial and temporal, between the subject and a threat, but it simultaneously hopes for a final reckoning where there will be no further loops, recursions, or reframings. When online text is the stage for debating the nature of the real – its quantities and its qualities – doomscrolling might represent an attempt to wrest events back into historical time, in which progress, even if that is just temporal distance rather than development, can be measured by a movement of a present event into the past. We will be anchored in time rather than drowned in it. But in being anchored rather than swimming within time’s stream, there is also the more troubling and apocalyptic hope for some, at least, not for a chronological progressive history which now seems inaccessible, but that the experiential grey time of ‘lost futures and future losses’ will finally come to an end in doom as destruction, even extinction.^31^ Accounts will have been settled once and for all and we, too, might ‘call time’ on the ‘slow cancellation of the future’ familiar from neoliberalism but significantly intensified in pandemic time.^32^

If doom constitutes both a gesture towards and hope for judgment, then scrolling pulls in the opposite direction in its suggestion of endlessness. Digital reading as rolling over with the eye and moving text up to meet the gaze does not demand the turning required of a codex book; it therefore pushes beyond that object’s obvious boundedness in space.^33^ Although all paper texts and codices have a potential endlessness, only limited by the actions of rolling and turning brought into contact with them, online reading as scrolling blurs the embodied contact with the material ends and breaks of texts. As Jack Hartnell has suggested in a historical framing of the idea of the continuous page, the unfurling of scrolls frequently has a ritual function that invokes an unbroken relationship to the past. But he also notes that the preference for scrolls in medieval Europe for keeping financial, administrative, and legal documents, had a more practical origin: ‘a roll consisting of glued membranes of unbroken parchment could be much more easily expanded to contain new information than the hermetically stitched and bound quires of a codex, allowing for the ready growth or contraction of working documents and records’.^34^ A scroll invokes the completion and continuity of an idea of history itself, or of tradition, or even of religious truth, but it can only do so by concealing its other immanent temporal qualities – its ends, beginnings, excisions and insertions, rereadings and recursions – just as the continuity of a digital network of links obscures its gaps, elisions, breaks, and obliterations (in the literal sense of writing over or striking out script).

The scroll’s endlessness is not one that sits securely in relation to historical or chronological time, continuity or totality, then, but speaks instead to an endless folding in of more time and more information within time. Scrolling, punctuated by a clicking which punctures the fabric of one textual space and stiches another into it, is a practice that uses time to create a topologically complex text that disrupts the linearity frequently taken to underpin a secure marking of time.^35^ Scrolling anxiously is a form of killing time that prorogues doom as destruction by seeming to produce ‘yet time’ in which things could unfold differently; or maybe it simply fills in for time’s weakened progression; but it cannot help but defer the possibility of judging sufficiently that one could ever call time on events and experiences. Doom-scrolling might also be figured as making grey time by gathering information and affects that have different sensations of intensity, but emerge in relation to an impeded, ruckled, unsatisfying, and paradoxically arousing and frightening present.

## Vigil(ance)

‘Rejoice in hope, be patient in tribulation, be constant in prayer’.

Romans, 12:12.

I sometimes find myself with the odd experience of moving my index finger upwards over the page of a codex book, as muscle memory overrides my sense of the materiality of my medium. As I am met with the stubborn insistence of print on page, which demands my eye move down rather than bringing words to come up to meet me, I feel a brief moment of shame. Why should I expect the world to come to me rather than being prepared to move towards it, with eyes scanning down pages and hands turning? Of course, if something is coming towards you, you might be more likely to feel it as engulfing. In a way that is likely linked to the unboundedness of digital scrolling, sometimes I lie in bed and see words disappearing upwards in mind’s eye. It is an experience that isn’t exactly anxious in terms of content, but it certainly has an uncomfortable, anxious form. The psychologist Jade Wu has suggested that ‘Generalised Anxiety Disorder is basically a Twitter feed of worries in your head’;^36^ and doomscrolling compulsively pulls an unbounded and overwhelming world towards you through movements that come to feel like fruitless rumination. It might also be linked to the exaggerated scanning behaviours of hypervigilance that work to detect activity and threat (how much, how close, how bad?), which are, in turn, associated with clinical presentations of Generalised Anxiety Disorder. Scanning; scrolling; waiting; checking. And repeat.

In much current Cognitive Behavioural Therapy – the talking therapy of choice for treating GAD in the UK’s NHS – there is a particular emphasis on turning the time of worry and rumination towards more productive ends. People living with a diagnosis of GAD are encouraged to set aside a specific and limited time for worrying, using the tendency to problem-solve to categorise worries into those that are unimportant, those that cannot be solved and can only be lived with, and those which are important and can be solved.^37^ Although such techniques can be very effective in managing anxiety and alleviating mental distress, they require significant disciplining of the use of time. And one can note that in echoing discourses of control, productivity, management, and efficiency, these CBT techniques reproduce, in therapeutic form, a version of the ‘time discipline’ that E. P. Thompson famously identifies as one of the central modes of shaping social relations to support the reproduction of capitalism. This ‘efficient husbandry of time’, to borrow Thompson’s phrase, seeks to correct the excessive and putatively maladaptive chronic worry and hypervigilance in GAD, as problem-solving activity and attentiveness become unproductive cycling and petrified, passive waiting.^38^ But there are other temporal traditions and ‘time disciplines’ through which worry, (hyper)vigilance, and indeed doomscrolling might be understood – other ways of understanding how care as worry, anxiety, and attention can be thought alongside one another. Indeed, there might be other ways of taking care of anxiety itself that would not simply render its seemingly excess concern more temporally efficient, but instead make something of its radically expanded mode of attention.

The oldest occurrences of ‘waiting’ in English associate it clearly with modes of attention – a link retained more obviously in the French *attendre* that comes to Middle English via Old French (*tendere*) to express the idea of stretching or actively applying one’s mind or energies to something. Waiting is derived from the Old North French *waitier*, meaning to ‘to watch, lie in wait for’, the Old High German *wahten*, ‘to watch’, and the Germanic wak, to be wakeful. Whether coolly calculating or hotly tensed, both early transitive and intransitive forms connect waiting to the activity of lying in wait, often with hostile intent – to watch out or watch for, wakefully. But watchful waiting has also been closely associated with religious practices of vigilance that see themselves as using attention to enact a care for the future. In his history of Western temporal understandings, Reinhart Koselleck describes how time in pre-modern Europe was figured through a Christian world view that invoked a ‘constant anticipation of the End of the World on the one hand and a continual deferment of the End on the other’.^39^ From monastic cultures of patience to the invention of tariffed penance as a physical space of Purgatory in the twelfth century,^40^ various temporal practices and ideas of waiting emerged in this period emphasising the importance of attending in what was imagined to be the ‘end times’ to the imminence of the final reckoning – the Last Judgment. As Benjamin H. Snyder describes it, a daily temporal ‘culture of vigilance’ developed in Christian monastic and particularly Benedictine life with a suggestive relationship to the etymological root of ‘waiting’. Benedictine monks, who created some of the first mechanical clocks in the Western world, indeed used the vigil (from the Latin *vigilia* – watchfulness, wakefulness, or even sleeplessness), to account for their practice of praying through the night to await the Second Coming of Christ and not be caught distracted or drifting. Vigilant waiting was ‘thus a primary site in which to demonstrate faith through patient watchfulness and persévérant wakefulness’.^41^ The vigil, though likely often painful to the self, was a way of taking care of time – of taking care of a relationship towards the future figured as the *eschaton* in which time itself would come to an end.

According to Koselleck, the inauguration of modernity signalled a shift away from prophecy – away from ideas of the future stabilised by the Last Judgment, when time moves into an eternity, the nature and structure of which is already known – towards an idea of prognosis in which human action can effect and create an unknown future by seizing historical time.^42^ By this account, doomscrolling might be said to fall between these two ideas of time. For although it partakes of vigilance, it is always excessive, slippery, repeating itself not in the confidence of a final judgment, but in the fear that events *cannot* be made to count or adhere. As we have seen, doomscrolling is also an attempt to wrest experience back into progressive, historical time and get things moving forwards; but its affect is often only one of ‘mechanical progression’, to use Minkowski’s terms – an illusion of forward movement in a time when progressive history feels like a threadbare fantasy. However, this is not to say that doomscrolling falls completely out of phase with what is sustaining. Doomscrolling has a certain attachment to hope and even the possibility of positive change. One can mark this in its signalling of a wish to be involved with and attached to a world of others; in its odd mimicking of a kind of conviviality and leisure; in its back and forth that echoes sustenance or conversation; and in its closeness to the forms of informational labour that for many now represent the world of work – a work that is as much about social reproduction as production.

Laden with contradiction, doomscrolling perhaps has a kinship with Lauren Berlant’s idea of ‘slow death’. Berlant writes in *Cruel Optimism* about particular modes of eating associated with obesity not simply as addicted and unhealthy, but as forms of lateral agency that have a particular, exhausted relationship to sovereignty and individual autonomy in neoliberal times when the idea of a progressive future has worn thin. This lateral agency is described as ‘floating sideways’, as body and life are experienced not only as ‘projects, but also sites of episodic intermission from personality […] inhabiting agency differently in small vacations from the will itself.^43^ Berlant does not represent this as healthy, but they acknowledge an orientation towards care in the desire to produce an intermission in conditions that feel intolerable in relation to a good-life that cannot be achieved. There is also an orientation towards what is sustaining in the potential for conviviality and nourishment always present within occasions of feeding and eating. Nevertheless, a radical ambivalence remains. For, to use Berlant’s terms, ‘slow death’ is a ‘zone of ordinariness’ ‘where life building and the attrition of human life are indistinguishable, and where it is hard to distinguish modes of incoherence, distractedness, and habituation from deliberate and deliberative activity’.^44^

Writing in 2011, Berlant makes an important distinction between the trauma-inflected rhetoric of crisis and what they call ‘crisis ordinariness’, or the slow living on in the ordinary time of neoliberalism which, in that historical moment, felt unchangeable in its grinding erasure of the future. COVID-19, which arrived following the shearing away of neoliberal consensus in the UK and the US, represents a shift in this time of the chronic. Most of the events that set people doomscrolling do indeed feel like crises: the progress of the pandemic; election results; takeovers and coups; climate catastrophe; shortages; the rationing of care. But it is becoming clear that even a pandemic figured as a crisis is not going to function as a straightforward historical marker or relief from the rhythms of the chronic. The fantasy that either interventions or laissez-faire strategies will bring a clear end to COVID-19 now seem like just that. Still, as optimism of all sorts is foreclosed, late capitalism is growing increasingly ineffective at distracting from the attrition of life that always undergirded it. As the pandemic forces a withdrawal from even cruel optimism, the reality of irreversible global extinctions caused by extractivism, alongside the ongoing violence against Black lives and the global poor, can no longer be so easily occluded or evaded.

One key to the process of rendering these realities visible has been the distribution of media technologies capable of turning the bystander – the one who waits and feels unable to act – into one who waits, vigilantly. In 1991, George Holliday videoed the ‘crisis ordinariness’ of a black man, Rodney King, being beaten by the Los Angeles Police Department. The footage later shown in court demonstrated the capacity for bystander video to provide powerful witness evidence, even if it did not lead easily to justice. As digital recording devices have become almost ubiquitous, people have increasingly turned the camera-eyes of their cell/mobile phones outwards to record in real-time – to capture and share the ongoing reality of catastrophe and premature and often violent death. Phone footage showed that Eric Garner (1970–2014) and George Floyd (1973–2020) between them uttered the words ‘I can’t breathe’ more than thirty times before their deaths under the weight of police brutality. The cell/mobile phone gives an account of the violence; it makes a record that can be used in legal proceedings, but can also be rapidly shared to enable broader social forms of judgment. Deaths that have previously been rendered ‘ungrievable’ are more easily accounted for – are made to count, as witnesses multiply.^45^

This instinct to turn the camera outwards, to press record and then share as the bystander becomes a lay, accidental witness who gathers a virtual crowd also called to witness, illuminates something, I think, about the compulsion to doomscroll. Discourses of healthcare heroism have been significant in the UK and the US during the pandemic because acting to deliver acute care registers in terms of crisis time –^46^ a time compatible with the markers of history. But lockdowns and living on with COVID-19 have demanded of populations whose labour has not been deemed ‘essential’ another kind of care that signifies less easily.^47^ This is the less active but nevertheless taxing work of remaining within the temporality of the chronic, of holding back, of waiting, and of becoming, in effect, a bystander. In such circumstances, those asked to wait may yet feel the need to act as a witness in order to signal their care and their attachment, using their digital device to record, even if only on the retina and in the mind, the tally of deaths, the ongoing violence, the attrition and loss. By making a record, even only within the psychic life of time rather than the temporality of history, realities witnessed at a distance might nevertheless be cared for and felt to count.

## Literary accounting

flowing and drawn, and since our knowledge is historical, flowing, and flown.

Elizabeth Bishop, ‘At the Fishhouses’.

Although *Cruel Optimism* is not a book centrally concerned with the literary, Berlant notes, as a literary scholar, that temporalities of slow death, of selfcontinuity rather than self-extension, don’t seem to fit with novel time. ‘[L]ives are not novels’, they state.^48^ As a literary scholar too, I have noted how the compulsion to doomscroll during the pandemic has interfered with the capacity to align my lived time with the forms of attention required to read literary fictions: the holding of multiple elements in mind and their projection into a future of resolution or even just an ending – a basic commitment to their onward pull. It seems damaging to the formal structure of most novels to scroll through them in a desultory back and forth, chopping and rolling, as if poised in a waiting-room ever-ready to be called to an appointment. The historical form of the novel created particular fictional spaces and reading practices that opened up in relation to new configurations of work and leisure, but these delineated arrangements are now under significant stress. The space and time of textual absorption is penetrated by demands that call for attention in ways that seem, for me at least, to break apart the affordances of the long form novel. Nevertheless, I did find myself able to read Don DeLillo’s very short fiction, *The Silence* (2020). Perhaps this was because of the text’s own formal work to trace the pulse of anxious reading and anxious textuality. Something about its rhythms matched my doomscrolling.

The novel is set in 2022 on the night of the Super Bowl. Jim Kripps, who is white (we are led to assume by the fact that he is not racially marked in the text), and his wife Tessa Berens, who is black, are flying from Paris to Newark. Their plane crash-lands as the world’s computer technologies are simultaneously arrested. Diane Lucas and her husband Max Stenner are waiting in their Manhattan apartment for Jim and Tessa to arrive for their Super Bowl party. One of Diane’s former physics students, Martin Dekker, is waiting with them. Part of the anxious formal affect of *The Silence* is the fact that, as Anne Enright has noted:

Information comes in packages, and it is hard to know what is important, what is not important, and what feels as though it was written by some ‘automated process’, one that writes catalogue copy for stuff you possibly need but don’t really understand.^49^

The book’s insistent, telegraphic prose in which distinctions are flattened out, invokes an anxious sense that there is little possibility left of choosing or understanding what is important without the algorithms that funnel attention in a digital world. Reality is experienced as a rush of data points, buffering and refreshing, but bringing scant hope of differentiation or understanding in terms of a hierarchising of information.

*The Silence’s* insistence on the form of the list can be read as an extension of the excess of information and paranoid linking found in a tradition of postwar US fiction, that spans Thomas Pynchon’s *Gravity’s Rainbow* (1973), David Foster Wallace’s *Infinite Jest* (1996), and DeLillo’s own evocations of hyperconnectivity in relation to the ‘Airborne Toxic Event’ of *White Noise* (1985). But the list is also a radical contraction of this digressive, encyclopaedic mode in the face of overload: information accumulates, but patterns or conspiracies don’t readily connect. There is instead a sense, to use one of the phrases from the book, of ‘our immersion in a single sustained overtone’.^50^ Here, too much information (usually a synonym for too much affect) ends up flattening out the technical distinction between information and noise. Instead, the text offers up ‘too much of everything from too narrow a source code’ in which, as in grey time, differences in kind become flattened out.^51^ But even the differences in intensity that mark the affects of digital life are now leaching away. Instead, words and experiences accumulate but are immediately distributed rather than signifying, adhering, and then passing. The ‘single sustained overtone’ is a balancing of accounts that writes out human modes of perceptual differentiation. It evokes and performs reading as scrolling over rather than a mode of absorption, penetration, attachment.

*The Silence* is concerned with a crisis where the world as it is has been disarticulated. But this crisis doesn’t feel quite like a turning point in the course of the disease; it is more like an intensification of the ongoing anxiety of slow death than the fearful, traumatic agency of crisis time:

Missiles are not soaring over the oceans, bombs are not being dropped from supersonic aircraft.

But the war rolls on and terms accumulate.

Cyberattacks, digital intrusions, biological aggressions. Anthrax, smallpox, pathogens. The dead and disabled. Starvation, plague and what else?

Power grids collapsing. Our personal perceptions sinking into quantum distance.

Are the oceans rising rapidly? Is the air getting warmer, hour by hour, minute by minute.^52^

This is fiction in which narrative assumes the form of doomscrolling. Everything is too close but cannot be penetrated and absorbed; everything is happening at the same time. There is an anxious attempt to settle accounts, but multiple possibilities remain in play. Jim’s job as a claims adjustor thus becomes a moment of textual irony; for it is precisely the ability to measure and adjust losses and articulate stable accounts of value that is suddenly unpicked and flattened out. Martin speculates that somehow the event must be linked to ‘[d]ata breaches […] Cryptocurrencies […] Money running wild. Not a new development. No government standard’.^53^ Of course, there is no gold standard to stabilise the financial system; but the substitution of valuable matter with trust – with securities or reputation – that defines a fiduciary relationship has been unpicked too and replaced with something that seems ominously, anxiously de-sutured from a human capacity to account for value.

In this grey landscape, this grey time, or what DeLillo calls the ‘insomnia of this inconceivable time’, the text understands that there might be a strange longing for a ‘War that we can see and feel./ Is there a shred of nostalgia in these recollections?’^54^ As we have seen, the blackness, or indeed the whiteness, of fear can indeed sometimes be imagined as a relief from ongoing anxiety, if it can be felt as a prompt to action. In *Ugly Feelings*, Ngai identifies anxiety as a something that comes to replace melancholia or depression as the male intellectual’s ‘signature sensibility’.^55^ For her, it has a fundamentally aversive quality that produces a form of distance from implicitly feminised modes of absorption and asignificance, even as it turns away and distances itself from even its own aversion.^56^ But one might also note in Ngai’s examples of anxiety as ‘the male knowledge-seeker’s distinctive yet basic state of mind’, an identification with a very particular form of agency, even if only lateral, that is racialised as white.^57^ This is not to say that only white people are anxious or doomscroll; but anxiety rather than fear in the face of proximate and imminent existential threat can also be a privilege that plays out across racial lines. Martin’s closing fantasy in the grey time he inhabits is of gaining some existential freedom: ‘it may be time for me to embrace a free death. *Freitod* […] Time to sit and be still’:^58^ ‘All my life I’ve been waiting for this without knowing it’.^59^ Martin imagines, perhaps like Martin Heidegger, that anxiety is an existential orientation to the world that would enable him to seize his own situation, his relationship to death and therefore to Being.^60^ But he is not one whose loss has been chronically adjusted to a fearful reality of being seized – a fearful reality of premature death.

On 5 June 2020, in the middle of the COVID-19 pandemic and 10 days after George Floyd was murdered by police officer Derek Chauvin in Minneapolis, Saidiya Hartman published a piece of writing online that retold W. E. B. Du Bois’s short story ‘The Comet’, while stitching in moments of interpretation and contextual expansion. This transgeneric piece of creative/critical writing was entitled ‘The End of White Supremacy, An American Romance’; and it was a response to the chronic crisis of violent and premature Black death in the United States. Du Bois’s short story was published in *Darkwater* in 1920, and Hartman’s essay revisits this speculative fiction about a catastrophic event first published in the wake of the 1918 influenza pandemic and the race-based atrocities of ‘Red Summer’ of 1919. As Hartman notes:

The influenza pandemic of 1918 does not appear [in *Darkwater*]. Perhaps because microbes seemed benign when compared with the bloodletting of the Red Summer. Or because for every year between 1906 and 1920, black folks in cities experienced a rate of death that equaled the white rate of death at the peak of the pandemic.^61^

For some, the mass destruction of lives was never a deviation from the way things go on.

Medieval and early modern people read comets as harbingers of crisis and change. Horatio in *Hamlet* understands the reappearance of the Ghost within a political crisis already set in motion; it is a ‘prologue to the omen coming on’ that might take the form of ‘stars with trains of fire and dews of blood’ and a moon ‘sick almost to doomsday with eclipse’.^62^ So it is, too, in Du Bois’s text, with New York poisoned by the gases held in the comet’s tail. A black man, Jim, survives this miniature apocalypse by being mistakenly shut in a bank vault. He emerges to find a white woman, Julia, who is seemingly the only survivor beside himself. Two New Yorks; two Jims; two catastrophes as things fall from the sky and time is ruptured; two inter-racial couples; two meditations on how things can suddenly be levelled and what might emerge in the wake. *The Silence* and ‘The Comet’ are separated by 100 years but are suggestive counterpoints for a discussion of how lives are understood to come to count in moments of reckoning.

In ‘The Comet’, we learn that Jim is sent down into the bank’s vault because he is, quite explicitly, ‘nothing’ – of no account. ‘It was too dangerous for more valuable men’, we hear, and judgment – fi*ducia* – follows the clear lines scored by a history of white supremacy: ‘Few ever noticed him save in a way that stung’.^63^ Jim finds himself shut in the vault, confined with shackled gold that squats ominously alongside him under the bank. As Hartman points out:

The crypt harbors the secrets, the disavowed knowledge and missing volumes on which the great financial edifice rests, the same history that has relegated Jim to the bowels of the earth […] In this sunken place, slavery is the thematic ground […] he is confronted with his origins and pricked by the realization, the uncanny feeling of an equivalence or doubling between the gold in the trunk and the Negro in the vault.^64^

When Jim re-emerges, he finds that the comet has killed most in New York. And he knows enough to trust how his survival will be made to count: ‘If they found him here alone – with all this money and all these dead men – what would his life be worth?’^65^ Jim is called ‘the Messenger’ in the story but endures beneath a ‘star’ that is not the harbinger of the birth of Christ. Instead, the comet signifies death’s approach, bearing an echo of the pandemic of 1918 in the idea of *influenza* as a disease outbreak imagined, in its medieval usage, to be caused by celestial *influence*.^66^ He finds himself in a time that resembles the Last Judgment where he and the surviving white woman, Julia, might become a new Adam and Eve in an unfallen world. Julia quotes Proverbs 22:2 as worldly differences are levelled: ‘The rich and the poor are met together […] The Lord is the Maker of them all’.^67^ But as survivors return to the city and find Julia alone with Jim, so does the usual model of accounting: ‘Where is he? Let’s lynch the damned –’.^68^ Jim survives to be saved or condemned by a white woman’s word.

Hartman’s essay models her method of ‘critical fabulation’, which enables her to go back to a previous text or history and suture back in accounts and materials that have been brutally excised from the record. More than simply recording, she makes creative use of a more continuous conceptual fabric of fact and fiction to stich back the history of the Red Summer of lynchings in 1919, alongside a history of premature Black death, into a recounting of Du Bois’s ‘The Comet’. Sometimes she uses Du Bois’s words; sometimes she adds other quotations from Black Thought; mostly, she glosses with her own words. But the text has the texture of a repetitive going back and going over, or sewing rather than seamlessly gluing or pasting material back into the scroll of history and literature and criticism. It is a way of making things count when they have been given little account. Whereas DeLillo’s *The Silence* describes ‘the single sustained overtone’ of a disaster produced by a technological modernity in which the human capacity for valuation has been unpicked, Hartman’s method stitches material back into scrolls as stores of information and experience – the accounts of the world – from a point of judgment that insists on measuring, calculating, and accounting for the disasters, the sins and losses, of the past. And the effect of such stitches in time is the revivification of a particular history and a certain reparation – the possibility of, and an orientation towards, another kind of future.

## Yet time/Jetztzeit

This storm irresistibly propels him into the future to which his back his turned, while the pile of debris before him grows skyward.

Walter Benjamin, ‘Theses on the Philosophy of History’.

Maybe it doesn’t even need stating that doomscrolling is a form of ‘paranoid reading’. But it is easy to forget that Eve Kosofsky Sedgwick’s influential essay on that topic, first published in 1997, begins with reading during a pandemic – the AIDS crisis – and the possibility of a conspiracy against the marginalised people most affected by it. Sedgwick’s response was to suggest that critique and the hermeneutics of suspicion are not the only or even the most important modes of reading produced by a pandemic. Interested in what knowledge and reading do rather than simply reveal, she follows the psychoanalyst Melanie Klein in differentiating between two positions formed in infant life, but that most people find themselves occupying and oscillating between: the ‘paranoid-schizoid’ and the ‘depressive’ position. Writing in 1946, Klein describes the child’s loss of full identification and psychical continuity with the primary object, usually the mother, as an inevitable part of development. But in order to preserve the idea of the good lost object that can be held internally and that gives a good feed, the child splits it spatially, keeping the good object away from a bad object that attacks both from the inside and from the external world, and is felt to be responsible for a bad feed and painful but inevitable experiences of frustration and loss. The movement from this spatialising ‘paranoid schizoid’ position to a ‘depressive position’ that recognises the aggression of the attack on the loved object and looks, remorsefully, to repair the damage done – to put it back together over and through time – signals for Klein an emergent capacity to understand the object in reality as separate and neither wholly good nor fully bad, but both good and bad.^69^

In Sedgwick’s description, Klein’s paranoid-schizoid position is ‘understandably marked by hatred, envy, and anxiety’, whereas the depressive position is an ‘anxiety-mitigating achievement’, a reparative process.^70^ Sedgwick argues, first, that the paranoid-schizoid position, as it becomes a paranoid reading, is anticipatory and produces a particular relationship to time: ‘The unidirectionally future-oriented vigilance of paranoia generates, paradoxically, a complex relation to temporality that burrows both back and forward: because there must no bad surprises, paranoia requires that bad news be always already known’.^71^ All is already known and already judged. Secondly, she suggests that paranoia is mimetic in that it ‘seems to understand only by imitation […] *Anything you can do (to me) I can do worse*, and *Anything you can do (to me) I can do first* – to myself’.^72^ If violence and its attendant humiliation cannot be stopped, they must not emerge as things that take the subject ‘*as a surprise*’.^73^ Sedgwick finds in paranoid reading the need to excise the shame of being exposed to the loss latent within an unknown future that haunts Klein’s paranoid-schizoid position; and this need drives the urge for the stability of critique, judgment, and accounting. Read alongside Freud, the anticipatory and mimetic elements of imagining and engaging with trauma proleptically, as if it were already known and as if there were ‘yet time to turn it aside’, braid the paranoid doomscrolling that attempts to judge and evade loss with the time of anxiety.

A paranoid reading of doomscrolling, or a reading of its paranoid elements, can understand it as an attempt to settle accounts that cannot be reframed or rewritten. Psychically, it strives to hold on to a particular and unalterable account in an information ecology in which trust about the nature of reality itself is profoundly fractured – all can be overwritten according to different points of view, with no stable boundary between fact and fiction. In an intensely monetised digital environment, for example, radical fungibility fuels concerns over how things can be accounted for. In DeLillo’s *The Silence*, cryptocurrencies are a backcloth that gestures towards a paranoia-inducing technological sublime; but the blockchain technologies that underpin currencies like Bitcoin are in fact a technical solution to the loss of *fiducia* and the ever-present and realistic, but also paranoid, possibility that records and accounts might be falsified. As Xiaowei Wang explains, a blockchain is a decentralised, public, digitally distributed ledger. Sets of records are mathematically chained to previous blocks of records using a ‘hashing function’, in which computers solve mathematical problems by running through combinations of numbers in a way that uses enormous amounts of computing power and electricity. Once this process is complete, the blocks are secured on the blockchain: ‘to falsify a record would mean having to redo all the work for subsequent blocks on the chain, requiring so much electricity and resources that falsification is disincentivized’.^74^

Like all financial accounting, then, blockchain is a form of record-keeping that also stabilises a linear version of time. In a blockchain, a distributed technical infrastructure rather than a single space holds centralised records, and machine code rather than natural language mediates and ensures trust in an environment that imagines bad actors and a tendency towards falsification against which people and systems must be protected. But whereas the tamperproof structures of blockchain are unreadable by end-users, doomscrolling, like turning the mobile/cell phone eye outwards and pressing record, might be understood as a more psychically legible attempt to settle accounts, to assert the place of facts and events within a chain of linear time, and to get time moving again within that chain, even as its repetitive circularity suggests the frailty of the strategy’s psychical effectiveness.

At one level, doomscrolling does mark how our attention is funnelled and monetised, subjected to forms of surveillance capitalism. The paranoid thought that our attention is shaped and driven by algorithms as we go back to scrolling, again and again, is hardly unrealistic.^75^ A digital feed is frequently felt to be a bad, unnourishing one. Nevertheless, it might also be possible to say that doomscrolling uses a latent animation and lateral agency within online reading, which is always more than simply searching; it uses some of reading’s pleasures and attachments to make new relationships to material and to make that material ‘count’ in a particular way. If reading always uses the capacity of grammar and syntax to bind experience within time, there might yet be something more aligned with Klein’s ‘depressive position’, something more reparative, to be found in doomscrolling’s production of ‘yet time’.

Walter Benjamin’s ‘Theses on the Philosophy of History’ (1940) describes a ‘homogeneous, empty time’ that might initially seem to match *The Silence’s* greyed-out ‘insomnia of this inconceivable time’, although Benjamin actually has in mind a version of progressive, teleological history to which some might yearn to return.^76^ But in his search for a non-Hegelian, non-developmentalist, one might say unchained conception of history, Benjamin looks to the possibility of another kind of time that he calls *Jetztzeit* [now time]. This shatters history as ‘telling the sequence of events like the beads of a rosary’.^77^

Instead, *Jetztzeit* emerges as the historical materialist brings fragments of history and the material world of the past into contact with the present to form new constellations of meaning – relationships that give light and articulate pattern. Against the grain of the dominant texture of history, Benjamin’s *Jetztzeit* works as a ‘flash’ [ein *Aufblitzendes*] – an explosive force of nowtime that can ‘blast open the continuum of history’ to reveal something of a transfigured world within worldly time.^78^ Past oppression, catastrophe, and unseen configurations of materiality – the hidden potentials of the everyday written out of history as teleology or progression – are suddenly vividly reanimated in an instant, in the ‘present as the “time of the now” which is shot through with chips of Messianic time’.^79^

In *The Time that Remains* (2000), Giorgio Agamben brings Benjamin’s *Jetztzeit* into dialogue with the Pauline Epistles, opening up a specific reading of messianic time as the temporality between the coming of the Messiah (Christ’s resurrection in Biblical time) and the Day of Judgment, or the moment when time moves into eternity. Although these two times are frequently conflated, Agamben makes clear that Benjamin’s theory of *weak* messianic time is the time of the end rather than the end of time. This messianic time is a waiting time – ‘*the time it takes for time to come to an end*’ – that is given in order that people might make an end in the knowledge of the immanence and imminence of the *eschaton*.^80^ As Agamben puts it:

The Messiah has already arrived, the messianic event has already happened, but its presence contains within itself another time, which stretches its *paraousia*, not in order to defer it, but, on the contrary, to make it graspable. For this reason, each instant may be, to use Benjamin’s words, the ‘small door through which the Messiah enters’.^81^

There is time yet. Indeed, it is precisely this ‘yet time’ that medieval chantry practices and liturgies for intercession use to care for the already dead. Such practices of care, both for the past and its sins and for the future of resurrection, open up a new time in the present in which past and future are folded into one another. Benjamin himself notes, in relation to history, that ‘nothing that has ever happened should be regarded as lost’, but ‘only a redeemed mankind receives the fullness of its past’.^82^ It is only at Judgment Day, Benjamin argues, that all can be accounted for; as a consequence, the historical whole is not representable in historical time – it cannot yet be resurrected. Nevertheless, Benjamin argues that in the protracted immanence of the meantime, glimpses or shards of the messianic can be found in *Jetztzeit* – in the white light of the flash. The time that remains is the time of anxious vigilance that oscillates between the ‘paranoia’ of judgment’s imminence and immanence, and a ‘reparative’ orientation in which there is ‘yet time’ to remake something that has been fractured and dispersed.

In her book *Enduring Time*, written just before the shifts in the seemingly endless grey chronicity of neoliberalism of 2016, Lisa Baraitser attends to the way in which contemporary neoliberal time might be figured as withdrawing from both historical or developmental time and from Benjamin’s *Jetztzeit.* Instead, she argues that neoliberal time is marked by ‘the temporality of *suspension*’ that produces ‘practices of suspending the self, suspending hope for change, and suspending an unfolding future’.^83^ In an argument drawing from the climate of Kleinian psychoanalysis and Afropessimism, Baraitser explains that this time is not orientated towards future judgment or transfiguration following the messianic event, but understands itself already to be in the wake of a disaster and withdrawal. As in both *The Silence* and ‘The Comet’, loss has already taken place; it is always too late. Nevertheless, something endures that can be described as ‘the time that persists through material and affective attachments (what remains)’, Baraitser insists.^84^ This time understands itself as containing, immanently, a permanent principle of connection, relationality, and dependence that cannot be severed, and that is reaffirmed not ‘in a flash’, but over and through time.^85^ This is a greyed-out world in which things are always already fallen, dispersed and disaggregated. It is a world that needs to be brought back together, or resutured, in Baraitser’s words, through the ordinary vigilance of caring practices that make and remake, reaffirming connection in the face of what has been lost.

One might say that Hartman’s work of critical fabulation, turned as it is towards the disasters of the past, has something of Benjamin’s *Jetztzeit* bound into it. It brings the lost catastrophes of history and the present together in a flash that makes something new, changing both ‘now’ and ‘then’ in ways that explode a form of specifically Black life out of an epoch that never wanted to sustain it. And yet, by consciously writing ‘in the wake’ of slavery, as Christina Sharpe would have it,^86^ Hartman’s work simultaneously pulls away from the flash of *Jetztzeit.* Poised between critical judgment and the unfinished and unfinishable reparative work of going back and going over, this critical fabulation withdraws from the idea that an ongoing disaster might be fully redeemed in the white light of the flash. By repeating and stitching, as if adding in pockets of textual time to make Du Bois’s ‘The Comet’ ‘count’ differently, Hartman’s essay instead effects a significant oscillation between a powerful critique of the occlusions of white supremacy that cannot ever be mitigated, and the ongoing, unfinishable labour of sustaining Black life and lives by re-relating that which has been severed, dispersed.^87^

My aim in placing doomscrolling alongside the critical restitching and reworking of Hartman’s text, is to try to bring to light elements of the practice that do not seem captured by the idea of addiction or the penetration of attention by algorithmic capitalism, even though doomscrolling hardly produces a new progressive history that one could bring up on one’s screen and that doesn’t tick upwards and away in the next moment. There is really no time to read it all, even if one exhausts all one’s time scrolling through it. Still, I want to claim that doomscrolling speaks to a desire to recognise and produce a time that could contain and make count relationships between selves, others and worlds, even amidst the overwhelming accumulation of data and the grey rubble of catastrophe that DeLillo describes and evokes. Paradoxically aligned with task of the historical materialist, as Benjamin saw it, doomscrolling might thus be framed as practice of care: of self-soothing, yes, but also of affirming a relationship with the world, of making things count, of taking the flattened landscape of overwhelming information and gathering it up into the container of a mind as a pocket of time.

Of course, this is a risky strategy in which the mind may be petrified or even split apart. In doomscrolling, it is indeed often easier to feel care as woe and lament than the care of ‘making and remaking the world’. The reparative does not write over or obliterate the paranoid in doomscrolling, nor is the opposite the case; instead, there is a significant oscillation between positions – an oscillation that might indeed animate its ongoing, unending forms of reading. Baraitser finds in the ways in which we endure time – ‘waiting, staying, persisting, delaying, repeating, preserving, […], maintaining’ –^88^ modes that function as practices of care that, to use her terms, also insist on taking care of what remains, including the time that remains. To understand doomscrolling as a way of enduring time, then, rather than simply killing it, is to bring to consciousness the ongoing temporal force of its tendency towards critique, even paranoia, which nevertheless exists alongside a reparative attempt to re-suture a relationship to a world through and as time, affirming the minimal attachment towards a future that is always immanent in any relationality, in any moving outwards from the self.

Doomscrolling might be framed, then, as an attempt to take care of time that acknowledges the withdrawal of a progressive future and that even replicates the loosening of social bonds that have not survived neoliberalism’s penetration of our life world. At almost the same time, however, it attempts to gather together and remake contact. We are told and may well feel that anxious doomscrolling is maladaptive in its paranoid distribution of attention and obsessive attachment and reattachment; for it is a reading practice that does link excessively. But COVID-19 has also brought to consciousness how the world is materially interconnected in ways that might have been ignored or deemed excessive in the past, but are suddenly made to count and register differently so that, for example, only radical practices of withdrawal can interrupt viral spread.^89^ Although there is a clear place for action, for treatments or vaccines, for a pragmatic problem-solving that has its own vital force, and indeed for critique, other temporalities beyond those of crisis action – and indeed other ontologies – might need to make themselves heard.

In ‘The Universal Right to Breath’ (2020), the philosopher Achille Mbembe responds to COVID-19 by bringing together its primary symptom of breathlessness with an understanding of breathing as a radically shared common ground. He argues that:

We have never learned to live with all living species, have never really worried about the damage we as humans wreak on the lungs of the earth and on its body. Thus, we have never learned how to die. With the advent of the New World and, several centuries later, the appearance of the ‘industrialized races,’ we essentially chose to delegate our death to others, to make a great sacrificial repast of existence itself via a kind of ontological vicariate.^90^

Humans forced to inhabit the status of non-being and non-human ‘others’ are the ones whose deaths have primarily serviced the engine of extraction, wealth creation, and the time of putative development. But within COVID-19 and climate catastrophe, conditions are emerging where losses will now be borne by ‘selves’ too. Mbembe indeed insists that unless we recognise a radically ‘universal right to breath’, the totality of life is in jeopardy.

Breathing, as Mbembe understands it, articulates an ontology grounded not in the independence of sovereign selves but in a commingling with others and with the world: with the oxygen and carbon dioxide in the atmosphere; with viral particles; and with the multiple figurations of a world that is more than human in its radical interconnectivity and always already forming networks of intra-action, as Karen Barad would have it.^91^ Making and remaking this universal right to breath requires learning to live in a time prepared to interrupt the temporalities of *telos* that have driven dominant versions of history; it requires a withdrawal from the time of development or, in Mbembe’s terms, ‘a *voluntary cessation, a conscious and fully consensual interruption.* Without which there will be no tomorrow’.^92^ Mbembe indeed asks for a hiatus in which time might be used to understand the reciprocal maintenance required for the social to exist and those relationships to the world in and through which we subsist. Mbembe asks for a time of waiting that might precede problem-solving, but would be figured by paying attention to a shared, commingled vulnerability – a time of tending in which there might be ‘yet time to turn aside’, carefully, one might say.

Although doomscrolling can be felt to detach us, carelessly, from our immediate surroundings, and can all too easily slip into the hyperconnectivity of paranoid reading and thinking, there also is in it a form of weak, fragile utopianism that is worth taking care of – a utopianism paradoxically present in the anxiety endemic to our world. In the anxious desire to produce ‘yet time’ there is a gesture of hope that the time that we have and the things that subsist within it might yet be attended to – be tended to. It may be true that anxiety is unproductive in terms of sorting worries into rational containers, but it is unproductive because it also insists in on caring excessively, on worrying away in such a way that new spatio-temporal relationships and new modes of responsibility and justice might yet be imagined. And perhaps, in this ‘yet time’, one might understand oneself to have more topologically complex, less sovereign, and more lateral and commingled relationships to the world, rather than just experiences of being overwhelmed or having one’s bodily and social systems and borders hijacked.

Alongside all the other things doomscrolling is – for it speaks powerfully to the painful reality revealed by the pandemic that the line between what is violence and what is care is sometimes virtually indistinguishable – we might then understand it as an attempt to attend to bonds that are increasingly broken in terms of traditional models of connection and kinship, but can be glimpsed and understood as necessarily connected in a world that is both human and more than human. The kind of waiting we undertake in doomscrolling speaks to what Baraitser calls the ‘*permanent* non-severance of selves, others, and institutions from what sustains them’ in a human frame,^93^ but, we might also say, to the radical commingling that is also the reality of intra-active relations with the more than human world – with data and algorithms and viruses and bodily fluids and flesh and air. Miming the possibility of an articulated and conscious relationship between the anxious and the depressive, between the puncture of critique and the suturing of reparation, the minimal gesture of the finger on the mousepad or phone, which tracks a pushing away from the self into the world, might just be read, then, not as a dismissive swiping left that severs a healthy relationship to time. Doomscrolling might yet be understood as a gesture that insists on the need to make time in which multiple relations count: relations to others, to information, to a world of exteriority that is both human and more than human, and to the future itself, which cannot and must not be a repetition of the catastrophes of the past.

## Notes

1 William Shakespeare, *Hamlet*, II, II.

2 *Oxford Languages: Words of an Unprecedented Year* (Oxford: Oxford University Press, 2020). Eve Kosofsky Sedgwick, ‘Paranoid Reading and Reparative Reading, or, You’re So Paranoid, You Probably Think this Essay is About You’, in *Touching Feeling: Affect, Pedagogy, Performativity* (Durham: Duke University Press, 2003), pp. 123–52.

3 See Lisa Baraitser, *Enduring Time* (London: Bloomsbury, 2017), p. 174.

4 Berenice Fisher and Joan Tronto, ‘Toward a Feminist Theory of Caring’, in Emily K Abel and Margaret K Nelson (eds), *Circles of Care: Work and Identity in Women’s Lives* (New York: SUNY Press, 1990), p. 40.

5 For a diagnosis of Generalized Anxiety Disorder, excessive anxiety and worry must have been occurring ‘more days than not for at least 6 months’. American Psychiatric Association, *Anxiety Disorders: DSM-5 Selections* (Arlington: American Psychiatric Association, 2016), p. 40.

6 American Psychiatric Association, p. 1. I am grateful to Alex Morden Osborne for our extended discussions on the nature of anxiety during the preparation of her PhD thesis: *Shaking, Breaking, Remaking: Anxiety in Contemporary American Literature, 1990-Present* (2021).

7 Joanna Bourke, *Fear: A Cultural History* (London: Virago, 2006), p. 190.

8 Eugène Minkowski, *Lived Time: Phenomenological and Psychological Studies*, trans. Nancy Metzel (Evanston: Northwestern University Press, 1970), pp. 87–8.

9 Minkowski, p. 186.

10 Minkowski, p. 187.

11 Henri Bergson, *Creative Evolution*, trans. Arthur Mitchell (New York: Dover, 1998), pp. 3–4.

12 Minkowski, p. 298.

13 Minkowski, p. 299.

14 Sigmund Freud, ‘Inhibitions, Symptoms and Anxiety’, *The Standard Edition of the Complete Psychological Works of Sigmund Freud*, vol. XX (1925–1926): 75–176, p. 165. The phrase ‘yet time’ is Strachey’s fairly straight translation of Freud’s ‘noch Zeit’.

15 Adrian Wells, ‘A Metacognitive Model and Therapy for Generalized Anxiety Disorder’, *Clinical Psychology and Psychotherapy*, 6 (1999), pp. 86–95, p. 89.

16 Wells, p. 89.

17 Sianne Ngai, *Ugly Feelings* (Cambridge: Harvard University Press, 2005), p. 210.

18 Leonard Woolf, *The Journey Not the Arrival Matters* (London: Hogarth Press, 1969), pp. 9–10.

19 Mathew Arthur, ‘Writing Pandemic Feels’, *Capacious: Journal for Emerging Affect Inquiry*, 2.1–2(2019–2020), pp. vi–xxiii, p. vi.

20 Rebecca Comay, *Grey on Grey: On the Threshold of Philosophy and Art*, eds. Aron Vinegar and Kamini Vellodi (Edinburgh: Edinburgh University Press, 2023) (forthcoming).

21 For a full articulation of the concept of grey time and anachromism, see Laura Salisbury, ‘Grey Time: Anachromism and Waiting for Beckett’, in Aron Vinegar and Kamini Vellodi (eds), *Grey on Grey: On the Threshold of Philosophy and Art* (Edinburgh: Edinburgh University Press, 2023) (forthcoming).

22 Algirdas Greimas and Jacques Fontanille, *Semiotics of Passion: From States of Affairs to States of Feeling* (Minneapolis: University of Minnesota Press, 1992), p. 9.

23 Greimas and Fontanille, p. 6.

24 Ngai, p. 246.

25 Bourke, p. 190.

26 Arthur, p. x.

27 *Hamlet*, I, V.

28 George P. Landow, *Hypertext: The Convergence of Contemporary Critical Theory and Technology* (Baltimore: Johns Hopkins University Press, 1992).

29 See, for example, Tristan Harris, ‘The Slot Machine in your Pocket’, *Der Spiegel*, 27 Jul 2016. https://www.spiegel.de/international/zeitgeist/smartphone-addiction-is-part-of-the-design-a-1104237.html

30 Matthew 13:49.

31 Rebecca Comay, *Mourning Sickness: Hegel and the French Revolution* (Stanford: Stanford UP, 2010), p. 142.

32 Franco ‘Bifo’ Berardi, *After The Future* (London: AK Books, 2011), pp. 18–19.

33 Although the word scroll is etymologically linked to scraps or shreds, it takes precedence over the word roll in the 1600s for texts and scripts, becoming particularly associated with deeds and bonds. As a verb, scrolling is linked with computer interfaces in 1971, as if a scroll were stretched vertically across a screen and could be rolled and unrolled to allow different parts of the surface to come into view.

34 Jack Hartnell, ‘The Continuous Page’, https://courtauld.ac.uk/research/research-resources/publications/courtauld-books-online/continuous-page-scrolls-and-scrolling-from-papyrus-to-hypertext/0-the-continuous-page-jack-hartnell/

35 For an account of Michel Serres’s topological theory of time and its relation to textuality, see Laura Salisbury, ‘Michel Serres: Science, Fiction, and the Shape of Relation’, *Science Fiction Studies*, 33.1(2006), pp.30–52.

36 Quoted by Jessica Klein, ‘Why does endlessly looking for bad news feel so strangely gratifying – and can we break the habit?’, 3 March 2021, https://www.bbc.com/worklife/article/20210226-the-darkly-soothing-compulsion-of-doomscrolling

37 Paul Farrand, Joanne Woodford, Faye Small, *Managing Your Worries* (Exeter: CEDAR/University of Exeter, 2019).

38 E. P. Thompson, ‘Time, Work-discipline, and Industrial Capitalism’, *Past and Present*, 38 (1967): 56–97, p. 78.

39 Reinhart Koselleck, *Futures Past: On the Semantics of Historical Time*, trans. Keith Tribe (New York: Columbia University Press, 2004), p. 11.

40 Jacques Le Goff, *The Birth of Purgatory*, trans. Arthur Goldhammer (Chicago: University of Chicago Press, 1981).

41 Benjamin H. Snyder, ‘From Vigilance to Busyness: A Neo-Weberian Approach to Clock Time’, *Sociological Theory*, 31.3 (2013), pp. 243–66, p. 252.

42 Koselleck, pp. 20–2.

43 Lauren Berlant, *Cruel Optimism* (Durham: Duke University Press, 2011), p. 116.

44 Berlant, p. 96.

45 Judith Butler, *Precarious Life: The Powers of Mourning and Violence* (London: Verso. 2004).

46 Stephanie Davies, ‘The politics of staying behind the frontline of coronavirus’, *Wellcome Open Research*, 5 (2020), p. 131.

47 The labour of many key workers has also registered less significantly in public discourse. Those who work in supermarkets or delivery drivers have been asked to intensify their chronic rhythms of labour rather than inhabit heroic crisis time.

48 Berlant, p. 99.

49 Anne Enright, https://www.theguardian.com/books/2020/oct/22/the-silence-by-don-delillo-review-the-machine-stops

50 Don DeLillo, *The Silence* (London: Picador, 2020), p. 14.

51 DeLillo, p. 105.

52 DeLillo, p. 77.

53 DeLillo, p. 85.

54 De Lillo, p. 78.

55 Ngai, p. 214.

56 Ngai, p. 246–7.

57 Ngai, p. 247. *The Silence* is part of an ongoing tradition in contemporary US fiction that frames anxiety alongside a crisis of white masculinity. A striking recent example is Nick Drnaso’s graphic novel, *Sabrina* (2018), which takes place in the wake of the eponymous character’s murder by a men’s rights activist. Despite the lurid qualities of this scene, and an aftermath of clickbait headlines and conspiracy theories, anxious distrust plays out in greyed-out, radically slowed square frames that mimic the undifferentiated strip-mall architecture with which the events are surrounded. Sabrina’s boyfriend, Teddy, and his friend, Calvin, inhabit a flat yet paradoxically hyper-vigilant state in which everything and nothing seems meaningful.

58 DeLillo, p. 102.

59 DeLillo, p. 103.

60 For Martin Heidegger on boredom see, *The Fundamental Concepts of Metaphysics: World, Finitude, Solitude.* Trans. William McNeill and Nicholas Walker (Bloomington: Indiana University Press, 1995).

61 Saidiya Hartman, ‘The End of White Supremacy, An American Romance’, https://bombmagazine.org/articles/the-end-of-white-supremacy-an-american-romance/

62 *Hamlet*, 1.1.

63 W. E. B. Du Bois, ‘The Comet’, *Darkwater* (New York: Washington Square Press, 2004), p. 195.

64 Hartman.

65 Du Bois, p. 197.

66 ‘Etymologia: influenza’, *Emerging Infectious Diseases*, 12.1(2006), p. 179.

67 Du Bois, p. 205.

68 Du Bois, p. 208.

69 Melanie Klein, ‘Notes on Some Schizoid Mechanisms’, *The International Journal of Psycho-analysis*, 27 (1946), pp.99–110.

70 Sedgwick, p. 128.

71 Sedgwick, p. 130.

72 Sedgwick, p. 131.

73 Sedgwick, p. 133.

74 Xiaowei Wang, *Blockchain Chicken Farm and Other Stories of Tech in China’s Countryside* (New York: Farrar, Strauss and Giroux, 2020), p. 59.

75 Shoshana Zuboff, *The Age of Surveillance Capitalism* (London: Profile Books, 2019).

76 Walter Benjamin, ‘These on the Philosophy of History’, in *Illuminations*, trans. Harry Zohn (New York: Harcourt and Brace, 1968), p. 261.

77 Benjamin, p. 263.

78 Benjamin, p. 262.

79 Benjamin, p. 263.

80 Agamben, p. 67.

81 Agamben, p. 71.

82 Benjamin, p. 254.

83 Baraitser, p. 161–2.

84 Baraitser, p. 166.

85 Baraitser, p. 166.

86 Christina Sharpe, *In the Wake: On Blackness and Being* (Durham: Duke University Press, 2016).

87 This method sits within a strand of contemporary US speculative literature that uses time-shifts to enable Black life to survive and even flourish within and alongside narratives of death and extinction. Nisi Shawl’s Afrofuturist *Everfair* (2016) explicitly revisions the past to avert the brutal deaths of millions caused by colonialism in the former Belgian Congo. Shawl materialises the possibility that being a good ancestor to those in the future might require looping back to remake historical time in a way that cares for ancestors in the past. They write back into the past, understanding that the trauma has already happened, but work as if there were ‘yet time’ to turn historical atrocity aside. Alexis Pauline Gumbs’s experimental *M Archive: After the End of the World* is a speculative grouping of documents ‘written from and with the perspective of a researcher, a post-scientist sorting artifacts after the end of the world’ (Durham: Duke University Press, 2018, p. xi). The textual fragments record the present of capitalist extractivism and antiblackness from an imagined far future in which Black experience has enabled both survival and a reshaping of the contours of the human.

88 Baraitser, p. 166.

89 Hinchliffe et al. describe the pandemic’s clear articulation of how planetary health must be taken up in collective ways, beyond relations as usually imagined. Stephen Hinchliffe, Lenore Manderson, and Martin Moore, ‘Planetary Healthy Publics After COVID-19’, *The Lancet*, April 2021, https://www.thelancet.com/journals/lanplh/article/PIIS2542-5196(21)00050-4/fulltext

90 Achille Mbembe, ‘The Universal Right to Breathe’, *Critical Inquiry*, 47, April 13, 2020, https://critinq.wordpress.com/2020/04/13/the-universal-right-to-breathe/. Lisa Baraitser has extended her thinking about temporal endurance in the light of Mbembe’s work in ‘The Maternal Death Drive: Greta Thunberg and the Question of the Future’, *Psychoanalysis, Culture and Society*, 25 (2020), pp. 499–517. https://link.springer.com/article/10.1057/s41282-020-00197-y

91 Karen Barad, *Meeting the Universe Halfway: Quantum Physics and the Entanglement of Matter and Meaning* (Durham: Duke University Press, 2007).

92 Mbembe.

93 Baraitser, p. 164.

